# A New Species of *Nanorana* (Anura: Dicroglossidae) from Northwestern Yunnan, China, with Comments on the Taxonomy of *Nanorana arunachalensis* and *Allopaa*

**DOI:** 10.3390/ani13213427

**Published:** 2023-11-06

**Authors:** Shangjing Tang, Shuo Liu, Guohua Yu

**Affiliations:** 1 Key Laboratory of Ecology of Rare and Endangered Species and Environmental Protection (Guangxi Normal University), Ministry of Education, Guilin 541004, China; sjtang163@163.com; 2 Guangxi Key Laboratory of Rare and Endangered Animal Ecology, College of Life Science, Guangxi Normal University, Guilin 541004, China; 3 Kunming Natural History Museum of Zoology, Kunming Institute of Zoology, Chinese Academy of Sciences, Kunming 650223, China

**Keywords:** *Nanorana*, *Paa*, *Chaparana*, *Allopaa*, northwestern Yunnan

## Abstract

**Simple Summary:**

Currently, the genus *Nanorana* contains thirty-two species, and four of them belong to the subgenus *Nanorana*, namely *N. bangdaensis*, *N. parkeri*, *N. pleskei*, and *N. ventripunctata*. In this study, on the basis of molecular and morphological evidence, we described a new species of *Nanorana* (*Nanorana*) from northwestern Yunnan, China, where only one member (*N. ventripunctata*) of *Nanorana* (*Nanorana*) has been reported. Additionally, the taxonomic status of *Nanorana arunachalensis* and *Allopaa hazarensis* were discussed, and subgeneric allocations of *Nanorana* species were suggested. The findings in this study bring the number of *Nanorana* species to 33 and improve our understanding on the taxonomy of genus *Nanorana* and the species diversity of *Nanorana* (*Nanorana*), an alpine group widely distributed in the southern and southeastern QTP.

**Abstract:**

The genus *Nanorana* contains three subgenera, namely *Nanorana*, *Paa*, and *Chaparana*, and currently, there are four species known to science in *Nanorana* (*Nanorana*). In this study, we describe a new species belonging to the subgenus *Nanorana* from northwestern Yunnan, China. Phylogenetically, the new species, *Nanorana laojunshanensis* **sp. nov.**, is the sister to the clade of *N. pleskei* and *N. ventripunctata*. Morphologically, the new species can be distinguished from known congeners by the combination of following characters: present tympanum, equal fingers I and II, small body size, yellow ventral surface of limbs, distinct vomerine teeth, indistinct subarticular tubercles, head width greater than head length, slender supratympanic fold, absent dorsolateral fold, nuptial spines present on fingers I and II in adult males, absent vocal sac, and paired brown spines on the chest. Moreover, we suggest moving the genus *Allopaa* into *Nanorana* (*Chaparana*) and consider that *N. arunachalensis* is neither an *Odorrana* species nor a member of the subfamily Dicroglossinae (therefore *Nanorana*), but probably represents a distinct genus closely related to *Ingerana* or belongs to *Ingerana*, pending more data. Additionally, we consider that *Nanorana minica* deserves the rank of an independent subgenus, and we suggest assigning *N. arnoldi*, *N. blanfordii, N. ercepeae, N. polunini, N. rarica, N. rostandi, N. vicina, N. xuelinensis*, and *N. zhaoermii* into the subgenus *Paa* and placing *N. kangxianensis*, *N. phrynoides*, and *N. sichuanensis* in the subgenus *Chaparana*.

## 1. Introduction

The Hengduan Mountains, located at the southeastern edge of the Qinghai–Tibetan Plateau (QTP) and having experienced major uplift between the late Miocene and the late Pliocene [1], are composed of a series of discrete north-to-south mountain ranges with alternating deep river valleys. They are characterized by extremely complex and diverse climatic and topographic conditions resulting in altitudinal zonation, which supports and isolates species inhabiting different niches [2] and has greatly contributed to the appearance of many new species [3]. As one of the 34 global biodiversity hotspots [4,5], the Hengduan Mountains are distributed with approximately 30% of the amphibian species of China and are particularly unusual in having the richest endemic alpine amphibian fauna-adapted cold conditions [6]. Furthermore, it has become a hotspot for the discovery of new species (e.g., [7,8,9]), indicating that the species diversity of amphibians in this area is still highly underestimated, and there are still cryptic amphibian species that have been detected but not described (e.g., [10]).

The genus *Nanorana* Günther, 1896 [11] is endemic to Asia. It has a wide distribution from the Himalayan region of northern Pakistan; northern India, Nepal; and western China through Myanmar, Thailand, Laos, and northern Vietnam to montane central and southern China [12]. Currently, it contains 32 species [12] and can be divided into three subgenera, namely subgenus *Nanorana*, subgenus *Paa*, and subgenus *Chaparana* [13], although some species were not included in the study of Che et al. [13]. Originally, the two subgenera *Paa* and *Chaparana* were erected as two independent genera by Dubois [14] and Bourret [15], respectively. Roelants et al. [16] revealed that *Nanorana* is imbedded within *Paa* on the basis of molecular data, and Jiang et al. [17] also provided molecular evidence for paraphyly of *Paa* with respect to *Nanorana* and the polyphyly of *Chaparana*. Subsequently, Chen et al. [18] placed *Chaparana* and *Paa* into *Nanorana* on the basis of a paraphyletic *Paa* with respect to *Nanorana* and *Chaparana*, and this was followed by Frost et al. [19], who placed *Chaparana* and *Paa* into the synonymy of *Nanorana* to resolve the paraphyly of *Paa* with respect to *Nanorana* (sensu stricto). Although Ohler and Dubois [20] presented a taxonomy of the tribe Paini in which *Chaparana* and *Nanorana* were treated as two independent genera and *Paa* was treated as a subgenus of *Chaparana*, it is difficult to address because they did not take into account previous molecular results [19] and recognized paraphyletic (genus *Chaparana* and its subgenus *Paa*) and polyphyletic (subgenus *Chaparana*) taxa [12]. Therefore, the placement of *Chaparana* and *Paa* into synonymy of *Nanorana* [18,19] was followed by most recent studies [13,21,22,23,24,25,26,27,28,29], although recently, Dubois et al. [30] presented a new classification of the tribe Paini in which *Nanorana*, *Paa*, *Chaparana*, *Feirana* Dubois, 1992 [31], and *Gynandropaa* Dubois, 1992 [31] remained independent, while two new genera (*Diplopaa* Dubois, Ohler, and Pyron, 2021 [30] and *Ombropaa* Dubois, Ohler, and Pyron, 2021 [30]) were erected.

*Nanorana* (*Nanorana*), which is widely distributed in the southern and southeastern QTP, is an alpine group within the family Dicroglossidae. Currently, it is composed of four species, namely *N. pleskei* Günther, 1896 [11], *N. parkeri* (Stejnger, 1927) [32], *N. ventripunctata* Fei and Huang, 1985 [33], and *N. bangdaensis* Rao, Hui, Zhu, and Ma, 2022“2020” [9]. In Yunnan, *Nanorana* (*Nanorana*) is known in three counties (Zhongdian, Deqing, and Weixi) located in the Three Parallel Rivers region, and only *N. ventripunctata*, a species occurring in lentic environments such as marshes, pools, and ponds at elevations ranging from 3120 to 4100 m, is recorded [34,35].

During field surveys in the sky-island mountains of the Three Parallel Rivers region, we collected some specimens belonging to *Nanorana* (*Nanorana*) from Mt. Laojun, Lijiang, northwestern Yunnan, China. Morphological comparisons and molecular phylogenetic analyses supported that these specimens are distinct from the four known species of *Nanorana* (*Nanorana*) and other members of genus *Nanorana*. Herein, we describe them as a new species of the genus *Nanorana*.

## 2. Materials and Methods

### 2.1. Sampling

**Figure 1:**
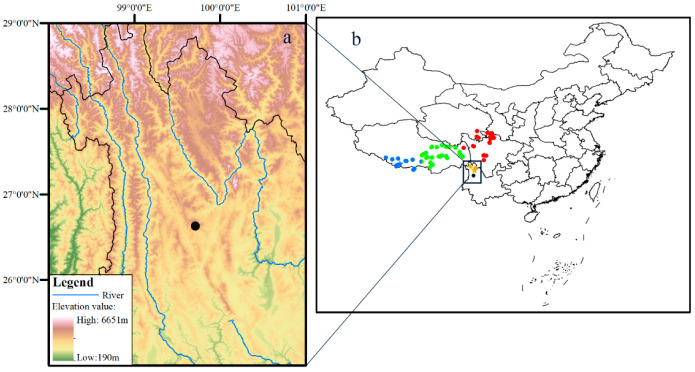
Map showing the type locality of *Nanorana laojunshanensis* **sp. nov.** in northwestern Yunnan, China (**a**) and known distribution localities of *N. parkeri* (blue), *N. pleskei* (red), *N. ventripunctata* (yellow), *N. bangdaensis* (green), and the new species (black) in China (**b**).

The classification of Frost [12] was followed for convenience. Specimens were collected at Mt. Laojun, Lijiang, Yunnan, China (Figure 1) by Guohua Yu in July 2019. Specimens were euthanized with ethyl acetate in a closed vessel and fixed and then stored in 75% ethanol. Liver tissues were preserved in 99.9% ethanol. All specimens were deposited at Guangxi Normal University (GXNU; Table 1).

### 2.2. Morphology

Morphometric data were taken using electronic digital calipers to the nearest 0.1 mm. The morphological terminology followed Fei et al. [36]. Measurements included the following: snout–vent length (SVL); head length (HL); head width (HW); snout length (SL); internarial distance (IND); interorbital distance (IOD); upper eyelid width (UEW); eye diameter (ED); nostril–eye distance (DNE); tympanum diameter (TD); forearm and hand length (FHL); tibia length (TL); foot length (FL); and length of foot and tarsus (TFL). Besides the specimens of the new species, eight specimens of *N. pleskei* and eight specimens of *N. ventripunctata* were also measured because phylogenetically, the new species is closer to these two species (see below). Comparative morphological data of congeners were taken from their original descriptions or re-descriptions [9,11,14,26,32,35,37].

Multivariate principal component analyses (PCAs) were conducted using SPSS 17.0 (SPSS Inc., Chicago, IL, USA) based on a correlation matrix of measurements. For these analyses, the measurements were corrected for size, and males and females were considered. Scatter plots of the first two PCA factors were used to examine the differentiation between the new species and its closely related relatives in the subgenus *Nanorana*.

### 2.3. Molecular Phylogenetic Analyses

Genomic DNA was extracted from liver tissue fixed in 99.9% ethanol using a standard phenol/chloroform protocol. We amplified and sequenced three mitochondrial genes (16S rRNA, COI, and cytb) and one nuclear gene (RAG-1). Primers used for PCR amplification and sequencing were obtained from previous studies [38,39,40] or designed by this study (see Table 2). PCR amplifications were performed in 25 µL reactions using the following cycling conditions: an initial denaturing step at 94 °C for 3 min; 35 cycles of denaturing at 94 °C for 60 s, annealing at 48–54 °C (51 °C for 16S, 48 °C for COI, 50 °C for cytb, and 55 °C for RAG-1), and extending at 72 °C for 60 s; and a final extending step of 72 °C for 10 min. The DNA sequences of both strands were obtained using the BigDye Terminator v.3.1 on an ABI PRISM 3730 following the manufacturer’s instructions. All new sequences have been deposited in GenBank under Accession Nos. OR671665–OR671686 and OR678554–OR678609 (Table 1). Sequences of known *Nanorana* species were obtained from GenBank. *Chrysopaa sternosignata* (Murray, 1885) [41], *Quasipaa boulengeri* (Günther, 1889) [42], *Allopaa hazarensis* (Dubois and Khan, 1979) [43], *Occidozyga lima* (Gravenhorst, 1829) [44], *Occidozyga lingnanica* Lyu and Wang, 2022 [45], *Occidozyga myanhessei* (Koehler, Vargas, Than, and Thammachoti, 2021) [46], *Ingerana borealis* (Annandale, 1912) [47], *Ingerana tenasserimensis* (Sclater, 1892) [48], *Hoplobatrachus chinensis* (Osbeck, 1765) [49], *Fejervarya cancrivora* (Gravenhorst, 1829) [44], *Sphaerotheca breviceps* (Schneider, 1799) [50], *Amolops mengdingensis* Yu, Wu, and Yang, 2019 [51], and *Odorrana hosii* (Boulenger, 1891) [52] were included as hierarchical outgroups.

DNA sequences were aligned using the MUSCLE option in MEGA version 7.0 [53] with the default parameters. Phylogenetic analyses were conducted for both 16S rRNA sequences and combined data of the four genes. For the analysis of combined data, the four gene alignments were defined using genes and codon positions, and then the best partitioning scheme and evolutionary models were estimated (Table 3) with PartitionFinder v.2.1.1 [54] using the “greedy” algorithm [55] for subsequent phylogenetic analyses. Bayesian phylogenetic analyses were performed in MrBayes v. 3.2. [56]. Two runs were performed simultaneously with four Markov chains starting from a random tree. The chains were run for 3,000,000 generations and sampled every 100 generations. The first 25% of the sampled trees were discarded as burn-in after the standard deviation of split frequencies of the two runs was less than a value of 0.01, and then the remaining trees were used to create a consensus tree and to estimate Bayesian posterior probabilities (BPPs). In addition, maximum likelihood (ML) analyses were conducted in raxmlGUI 2.0 [57] with 1000 rapid bootstrap replicates.

## 3. Results

### 3.1. Molecular Phylogeny

**Figure 2:**
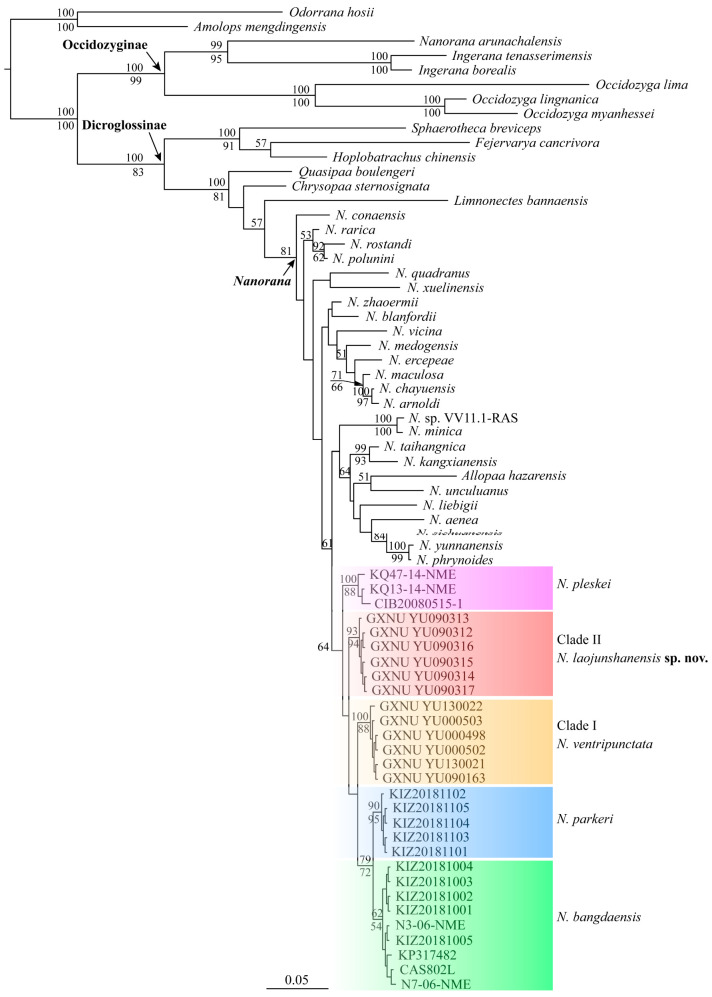
Bayesian phylogram of the genus *Nanorana* inferred from 16S rRNA sequences. Numbers above and below branches are Bayesian posterior probabilities and ML bootstrap values, respectively (only values above 50% are shown).

**Figure 3:**
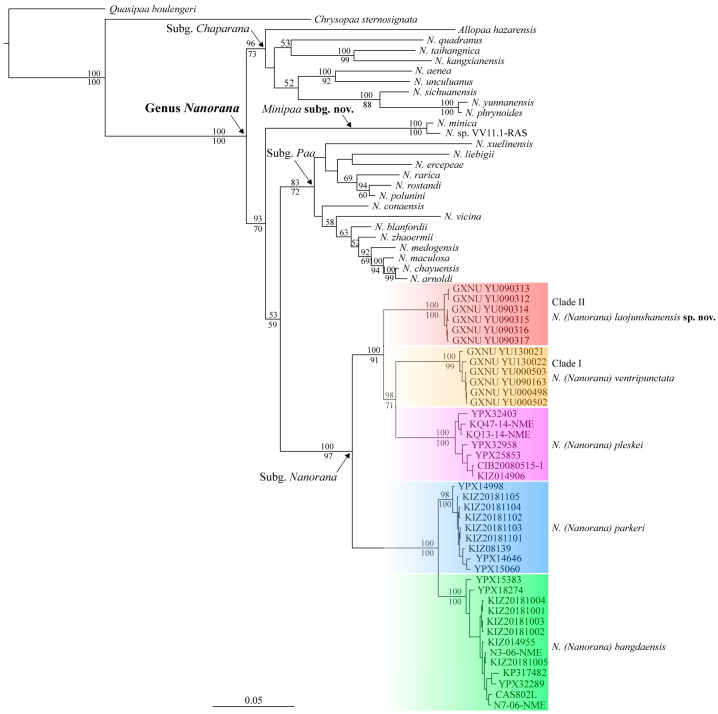
Bayesian phylogram of the genus *Nanorana* inferred from combination of 16S rRNA, COI, cytb, and Rag-1 sequences. Numbers above and below branches are Bayesian posterior probabilities and ML bootstrap values, respectively (only values above 50% are shown).

The aligned sequences of 16S rRNA, COI, cytb, and RAG-1 were 575 bp, 674 bp, 965 bp, and 1189 bp, respectively. Both phylogenetic analyses for 16S rRNA sequences and phylogenetic analyses for the combined data based on the best partitioning scheme and models revealed that *Nanorana* (*Nanorana*) from Yunnan consists of two distinct lineages (Figure 2 and Figure 3), one containing individuals from Zhongdian Plateau, known as *N. ventripunctata* (Clade I), and one containing samples from Mt. Laojun, Lijiang (Clade II). *Nanorana ventripunctata* (Clade I) was recovered as the sister to *N. pleskei*, and Clade II was recovered as the sister to the clade consisting of *N. ventripunctata* and *N. pleskei*. Genetic distances (p-distance) between the new lineage and known species of *Nanorana* (*Nanorana*) ranged from 1.6% (vs. *N. ventripunctata*) to 2.0% (vs. *N. bangdaensis*) in 16S, which is roughly equal to the distances between other species of subgenus *Nanorana*, and from 7.4% (vs. *N. ventripunctata*) to 10.6% (*N. bangdaensis*) in COI, which is greater than the distance between *N. bangdaensis* and *N. parkeri* (Table 4).

The specimen under the name *Nanorana arunachalensis* (Saikia, Sinha, and Kharkongor, 2017) [58] in GenBank did not cluster together with the subfamily Dicroglossinae (therefore *Nanorana*) but was nested in the subfamily Occidozyginae and was closer to *Ingerana* with strong supports (Figure 2). The sample of *A. hazarensis* was nested in the genus *Nanorana* and grouped together with members of the subgenus *Chaparana* with strong supports (Figure 3).

### 3.2. Morphometric Analyses

**Figure 4:**
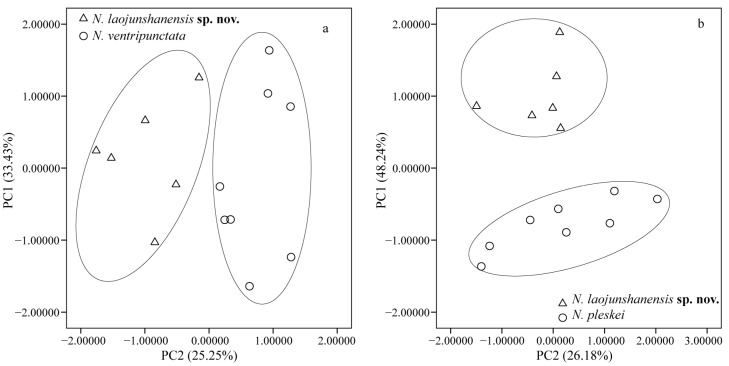
Scatterplots of principal components 1 and 2 of morphometric data of *N. laojunshanensis* **sp. nov.** and its two relatives, *N. ventripunctata* (**a**) and *N. pleskei* (**b**).

Morphological measurements of the new species are presented in Table 5, and the measurements of *N. pleskei* and *N. ventripunctata* examined in this study are presented in Tables S1 and S2. For the PCA analysis on the new species and *N. ventripunctata*, the first two principal components accounted for 58.68% of the total variance (Table 6), the loadings for PC2 were heavily loaded on UEW, ED, and NED (loading factor > 0.7), and differentiation between the new species and *N. ventripunctata* was found along the PC2 axis (Figure 4a), indicating that the new species differs from *N. ventripunctata* by its narrower upper eyelid, larger eye, and greater nostril–eye distance. For the PCA analysis on the new species and *N. pleskei*, the first principal component (PC1) accounted for 48.24% of the total variance, the loadings for PC1 were heavily loaded on IND, UEW, FHL, TL, TFL, and FL, and obvious differentiation was found along the PC1 axis (Figure 4b), indicating that the new species is different from *N. pleskei* by its narrower internarial distance, narrower upper eyelid, and longer limbs.

### 3.3. Taxonomic Account

The results of molecular phylogenetic and morphological analyses indicated that the specimens from Mt. Laojun, Lijiang, represent a distinct lineage, and it can be distinguished from its congeners by body size and the combination of texture and coloration pattern. Therefore, we describe it here as a new species.

**Figure 5:**
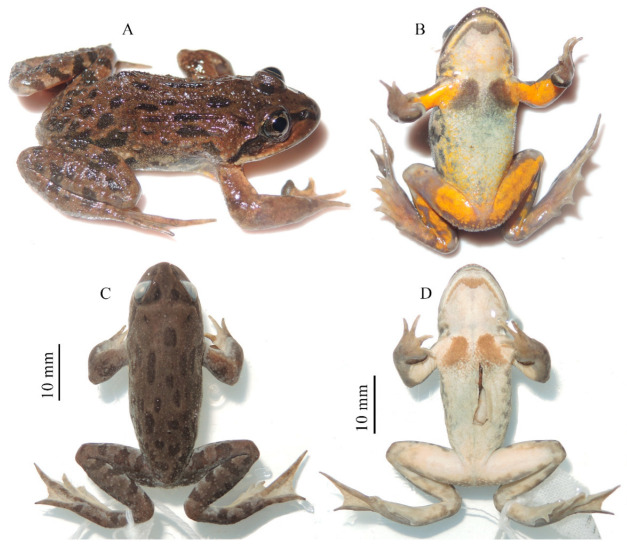
Holotype of *Nanorana laojunshanensis* **sp. nov.** in life (**A**,**B**) and in preservative (**C**,**D**).

**Figure 6:**
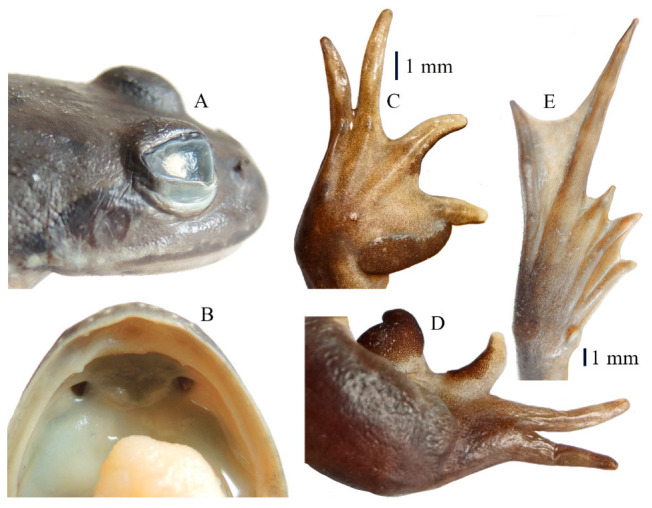
Views of tympanum (**A**), vomerine teeth (**B**), hand (**C**), nuptial pad (**D**), and foot (**E**) of the holotype of *Nanorana laojunshanensis* **sp. nov.**

**Figure 7:**
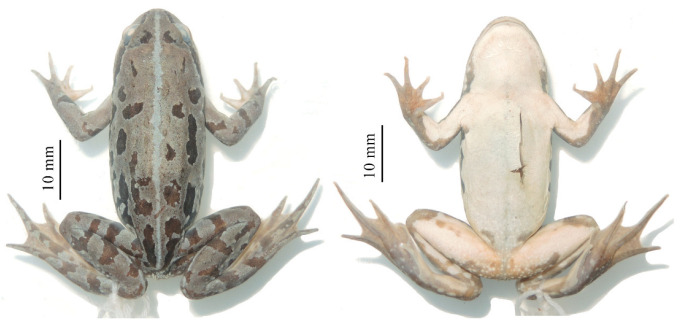
Dorsal and ventral views of the paratype of *Nanorana laojunshanensis* **sp. nov.** (GXNU YU090312) in preservative.

*Nanorana* (*Nanorana*) *laojunshanensis* **sp. nov.** (Figure 5, Figure 6 and Figure 7; Table 5)

http://zoobank.org/urn:lsid:zoobank.org:act:D44F3AC5-A50D-4908-A424-F4EFA512D209 (accessed on 17 August 2023).

Holotype: GXNU YU090314, adult male, collected on 24 July 2019 by Guohua Yu at Mt. Laojun, Lijiang, Yunnan, China (26°37′ N, 99°42′ E, 3982 m a.s.l.).

Paratypes: Four adult males (GXNU YU090313 and 090315–090317) and an adult female (GXNU YU090312) collected from the type locality at the same time by Guohua Yu.

Etymology: The specific epithet is named after the type locality, Mt. Laojun, Lijiang, Yunnan, China. We suggested “laojunshan slow frog” for the common English name and “老君山倭蛙 (Lǎo Jūn Shān Wō Wā)” for the common Chinese name.

Diagnosis: The new species was assigned to *Nanorana* (*Nanorana*) using the following morphological characters: oval tongue, shallowly notched posterior; blunt finger and toe tips; absent webbing between fingers, absent supernumerary tubercle; developed webbing between toes; paired patches of spine on chest [37]. The new species can be distinguished from other members of *Nanorana* by having a combination of the following characters: (1) present tympanum; (2) small body size; (3) distinct vomerine teeth; (4) indistinct subarticular tubercles; (5) head width greater than head length; (6) slender supratympanic fold; (7) finger I equal to finger II; (8) absence of dark blotches on ventral surface and yolk-yellow ventral surface of limbs; (9) absent dorsolateral fold; (10) nuptial spines present only on fingers I and II in adult males; (11) absent vocal sac; and (12) paired brown spines on chest.

Description of holotypes: Adult male (SVL 36.1 mm; Table 5); head width (HW 11.3 mm) greater than head length (HL 10.7 mm); rounded snout, slightly protruding beyond lower jaw in ventral view; distinct canthus rostralis; sloping loreal region; nostrils are oval, lateral, and nearer to the eye; interorbital distance (IOD 1.9 mm) is smaller than the internarial distance (IND 2.3 mm) and smaller than the upper eyelid width (UEW 2.4 mm); a small and indistinct pineal spot between the eyes; horizontal oval pupil; small, rounded tympanum (TD 1.3 mm), smaller-than-half eye diameter (ED 3.9 mm); slender, distinct supratympanic; distinct vomerine teeth in two short oblique rows between the choanae; oval choanae; oval tongue; shallowly notched posterior; no vocal sac.

Forelimbs are robust; length of fingers I and II is nearly equal, relative length of fingers I ≈ II < IV < III; fingertips are blunt, not expanded; webbing between fingers is absent; subarticular tubercles are indistinct; supernumerary tubercles at base of fingers are small and indistinct; inner metacarpal tubercle is flat, outer metacarpal tubercle is indistinct.

Hindlimbs are robust, with the tibiotarsal articulation reaching the posterior edge of the eye when the hindlimb is stretched alongside the body; the heels meet when the legs are positioned at right angles to the body; the length of toes III and V is nearly equal, relative length of toes I < II < III ≈ V < IV; all toe tips are blunt, not expanded; toes are fully webbed, webbing formula I0–1II0–1III0–1IV1–0V; subarticular tubercles are indistinct, formula 1, 1, 2, 3, 2; inner metatarsal tubercle is oval and small; outer metatarsal tubercle is absent.

Skin is rough with a longitudinal skin ridge on the anterior part of the dorsum and scattered large tubercles on the posterior part of the dorsum; many large tubercles are on the dorsal surface of the hindlimbs; the dorsal surface of the forelimbs is smooth; a few tubercles are on the body flanks. The ventral surface is relatively smooth; many white tubercles are around the vent.

Color of holotype in life: Dorsal surface of body, body flanks, and dorsal surface of limbs are brown mottled with irregular dark patches; side of head is grayish brown, with a dark band on the canthus rostralis from the tip of the snout to the anterior border of the eye and a dark band below the supratympanic fold. Ventral surface is grey white, with yolk-yellow patches on the ventral surface of limbs; iris is black with golden brown mottling.

Color of holotype in preservative: dorsal surface is faded to grayish brown, mottled with dark patches, pattern as in life; ventral surface is white.

Sexual dimorphism: Males possess nuptial pads with dense small brown spines on the base of fingers I and II and an extremely developed nuptial pad on the base of finger I (Figure 6D), as well as paired “/ \”-shaped patches of small brown spines on the chest. In addition, males have a M-shaped patch of brown spines on the edge of the lower jaw.

Variations: The female individual (GXNU YU090312) has a mid-dorsal vertebral stripe running from the posterior of the snout to the vent and a more smooth dorsal surface without conical spines (Figure 7).

Distribution and ecology: Currently, the new species is only known from the type locality and inhabits marshes and ponds. Adult males have nuptial pads and nuptial spines, suggesting that the breeding season of the new species is about July and August. No tadpoles were collected for the new species.

**Figure 8:**
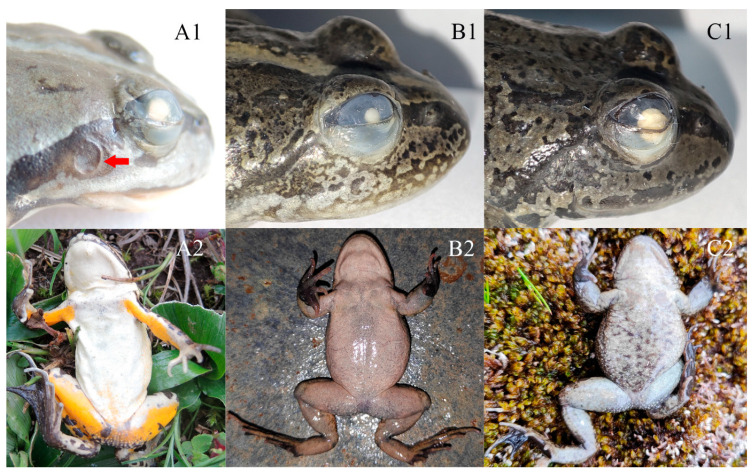
Tympanum region and ventral surface of *N. laojunshanensis* **sp. nov.** (**A1**,**A2**), *N. bangdaensis* (**B1**,**B2**), and *N. parkeri* (**C1**,**C2**). Tympanum of the new species is highlighted with arrow.

**Figure 9:**
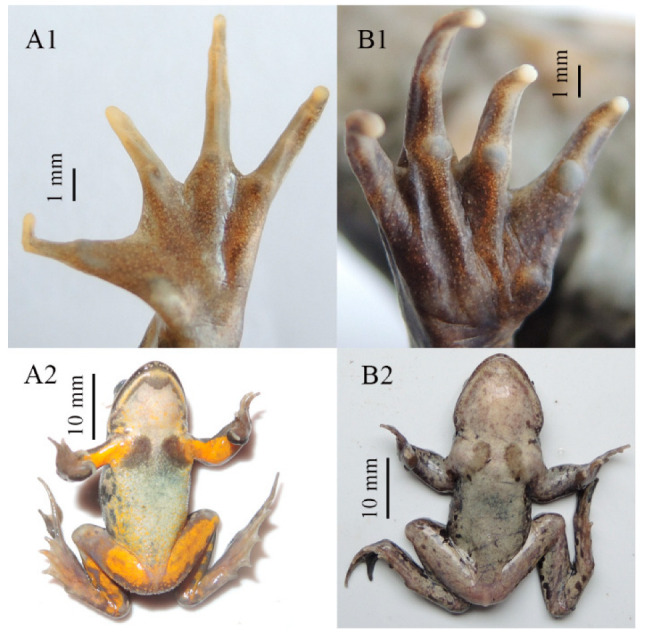
Hand and ventral surface of *N. laojunshanensis* **sp. nov.** (**A1**,**A2**) and *N. ventripunctata* (**B1**,**B2**).

Comparison: *Nanorana* (*Nanorana*) contains four species, namely *N. pleskei*, *N. parkeri*, *N. ventripunctata*, and *N. bangdaensis*. The new species can be distinguished from *N. parkeri* by having a tympanum (vs. absent; Figure 8), equal fingers I and II (vs. finger I longer than finger II), smaller body size (male SVL 33.3–38.5 mm and female SVL 42.9 in *N. laojunshanensis* **sp. nov.** vs. males 40–51 mm and females 50.1–51.5 mm in *N. parkeri*), yellow ventral surface of limbs (vs. greyish white; Figure 8), and distinct vomerine teeth (vs. absent or extremely weak). It can be distinguished from *N. pleskei* by its greyish brown dorsal surface (vs. olive green, yellowish green, or dark green), equal fingers I and II (vs. finger II longer than finger I), distinct vomerine teeth (vs. absent or extremely weak), and narrower internarial distance, narrower upper eyelid, and longer limbs (Table 6 and Figure 4). It can be distinguished from *N. ventripunctata* by indistinct subarticular tubercles (vs. distinct; Figure 9), a greyish white throat and belly with no dark patches and yolk-yellow ventral surface of limbs (vs. greyish white ventral surface scattered with dark blotches on the throat, belly, and/or ventral surface of limbs; Figure 9), and a narrower upper eyelid, larger eye, and greater nostril–eye distance (Table 6 and Figure 4). It can be distinguished from *N. bangdaensis* by a present tympanum (vs. absent; Figure 8), nuptial pads present on fingers I and II (vs. only finger I), a head width greater than head length (vs. head width equal to head length), a very rough dorsal surface with a dense longitudinal skin ridge on the dorsum and many tubercles on both the dorsum and the dorsal surface of the hindlimbs (vs. a smooth dorsal surface with only a few skin ridges on the dorsum), equal fingers I and II (vs. finger I longer than finger II), large black blotches on the flank (vs. many small spots on the flank), dark bands on the limbs (vs. absent), a white venter and yolk-yellow ventral surface of limbs (vs. beige), and a slender supratympanic fold (vs. thick).

The new species can be distinguished from *N. aenea* (Smith, 1922) [59], *N. annandalii* (Boulenger, 1920) [60], *N. gammii* (Anderson, 1871) [61], *N. liebigii* (Günther, 1860) [62], *N. minica* (Dubois, 1975) [14], *N. polunini* (Smith, 1951) [63], *N. rarica* (Dubois, Matsui, and Ohler, 2001) [64], *N. rostandi* (Dubois, 1974) [65], *N. unculuanus* (Liu, Hu, and Yang, 1960) [66], and *N. vicina* (Stoliczka, 1872) [67] by the absent dorsolateral fold (vs. present); from *N. arnoldi* (Dubois, 1975) [14], *N. maculosa* (Liu, Hu, and Yang, 1960) [66], *N. yunnanensis* (Anderson, 1879) [68], and *N. zhaoermii* Qi, Zhou, Lu, and Li, 2019 [22] by nuptial spines which are present only on fingers I and II in adult males (vs. present on fingers I–III); from *N. arunachalensis* by the greyish brown dorsal surface (vs. green) and small body size (female SVL 42.9 mm vs. female SVL 81.33 mm in *N. arunachalensis*); from *N. blanfordii* (Boulenger, 1882) [69], *N. chayuensis* (Ye, 1977) [70], *N. conaensis* (Fei and Huang, 1981) [71], *N. feae* (Boulenger, 1887) [72], *N. medogensis* (Fei and Ye, 1999) [73], *N. mokokchungensis* (Das and Chanda, 2000) [74], *N. phrynoides* (Boulenger, 1917) [75], and *N. sichuanensis* (Dubois, 1987) [76] by the absent vocal sac (vs. present); from *N. kangxianensis* (Yang, Wang, Hu, and Jiang, 2011) [77], *N. quadranus* (Liu, Hu, and Yang, 1960) [78], and *N. taihangnica* (Chen and Jiang, 2002) [79] by nuptial spines which are present on fingers I and II (vs. nuptial spines which are only present on finger I in *N. kangxianensis* and nuptial spines which are absent in *N. quadranus* and *N. taihangnica*); and from *N. xuelinensis* Liu, Zhang, and Rao, 2021 [26] by having paired brown spines on the chest (vs. black spines which are present on the chest, belly, lateral body, etc).

## 4. Discussion

Currently, four species are recognized in the subgenus *Nanorana (Nanorana)*, and only one is recorded in Yunnan, China [35]. In this study, morphological comparison and phylogenetic analyses based on mtDNA and nuDNA revealed that the populations from the east side of Jinsha River represent a new species that is the sister to the clade of *N. ventripunctata* and *N. pleskei*, bringing the species number of the subgenus *Nanorana* to five. It is worthwhile to further investigate the distribution boundary and species diversity of the subgenus *Nanorana*. *Nanorana bangdaensis* had been confused with *N. parkeri*. Zhou et al. [80] revealed that *N. parkeri* consists of two major lineages (lineages E and W) based on COI sequences. Moreover, recent comprehensive analyses based on whole genomic data [81] found substantial genomic isolation between the E and W lineages with highly restricted gene flow in a narrow geographic zone lying between them and suggested that endogenous selection is a dominant factor resulting in speciation between the W and E lineages. The type localities of *N. parkeri* and *N. bangdaensis* are Tingri, Tibet, China, and Bangda, Baxoi, Tibet, China, respectively. Therefore, the west lineage (W) refers to *N. parkeri* because it contains topotypes of *N. parkeri*, whereas the east lineage (E) represents *N. bangdaensis* because it contains topotypes of *N. bangdaensis* (Figure 1). The 16S distances between members of subgenus *Nanorana* range from 1.0% to 2.8%, smaller than the value of 3% as defined by Vieites et al. [82], but morphologically, they can be easily distinguished from each other. For instance, *N. bangdaensis* and *N. parkeri* differ from *N. pleskei*, *N. ventripunctata*, and *N. laojunshanensis* **sp. nov.** by the absent tympanum (vs. present), *N*. *bangdaensis* differs from *N. parkeri* by the nuptial pad present on the base of finger I (vs. present on both fingers I and II), and *N. laojunshanensis* **sp. nov.** can be distinguished from *N. ventripunctata* by the yolk-yellow ventral surface of limbs and the indistinct subarticular tubercles (Figure 9). Furthermore, the distributions of these five species are nearly discrete (Figure 1), and it has been revealed that gene flow between *N. parkeri* and *N. bangdaensis* is highly restricted [81], as mentioned above. Therefore, we consider that the five species of subgenus *Nanorana* are all valid. Generally, genetic distance between recently diverged species is relatively small (e.g., species of the *Amolops mantzorum* group; [83]). This is the same with the subgenus *Nanorana*, which started to diverge into different lineages ca. 3.7 Mya according to Hofmann et al. [23].

Additionally, the taxonomic status of *N. arunachalensis* needs further examination. It was originally placed in *Odorrana* by Saikia et al. [58] and was later transferred to *Nanorana* by Qi et al. [22] based on morphological characters. Most recently, Hofmann et al. [84] suggested excluding *N. arunachalensis* from *Nanorana* and reassigning it to the genus *Odorrana* based on a 16S phylogeny and genetic distances. However, this taxonomic change is unreliable in that the 16S phylogeny in Hofmann et al. [84] only contained members of Dicroglossinae and actually did not show any phylogenetic evidence that *N. arunachalensis* belongs to *Odorrana*. The two vouchers of nominal *N. arunachalensis* (ZSIS-M37 and ZSIS-M40) were sequenced by Saikia and colleagues and came from the type locality. In this study, our phylogenetic analyses revealed that the voucher of *N. arunachalensis* is nested in Occidozyginae and is closer to *Ingerana* with strong supports. Morphologically, *N. arunachalensis* is similar to *Ingerana* in having small finger and toe discs, a prominent supra-tympanic fold, an indistinct or hidden tympanum, and a dark inter-orbital band, according to Saikia et al. [58], Fei et al. [34], and Zug [85]. Therefore, assuming that these two vouchers of *N. arunachalensis* (ZSIS-M37 and ZSIS-M40) were identified correctly by Saikia and his colleagues, the phylogenetic analyses in this study indicate that *N. arunachalensis* probably belongs to *Ingerana* or represents a new genus closely related to *Ingerana* rather than *Odorrana* or *Nanorana*. Recently, Wangyal et al. [86] reported the first record of *N. arunachalensis* from Bhutan based solely on photographs. However, this record should be treated with caution and needs further investigation because it obviously differs from the types of *N. arunachalensis* by its brown dorsal surface (vs. green) and the absence of dark bands on the dorsal surface of the limbs. Thus, more studies employing molecular and morphological data are necessary to solve the taxonomy of *N. arunachalensis*.

Che et al. [13] divided the genus *Nanorana* into three subgenera, but some members of this genus were not included and therefore were not assigned to a subgenus by them. In this study, most known species of the genus were included, and phylogenetic analyses based on the combined data revealed that *Nanorana* contains four major lineages, three of which correspond to the three subgenera (Figure 3). *Nanorana arnoldi*, *N. blanfordii*, *N. ercepeae*, *N. polunini*, *N. rarica*, *N. rostandi*, *N. vicina*, *N. xuelinensis*, and *N. zhaoermii* were nested in the clade containing known members of the subgenus *Paa* (*N. liebigii*, *N. conaensis*, *N. medogensis*, *N. maculosa*, and *N. chayuensis*; Che et al. [13]). So, we suggested placing these nine species into the subgenus *Paa*. *Nanorana kangxianensis*, *N. sichuanensis*, and *N. phrynoides* form a clade with known members of the subgenus *Chaparana* (*N. quadranus*, *N. taihangnica*, *N. aenea*, *N. unculuanus*, and *N. yunnanensis*; Che et al. [13]), suggesting that these three species belong to the subgenus *Chaparana* (Table 7). *Nanorana minica* was once placed in same subgenus with members of *Paa* by Ohler and Dubois [20]. However, molecular phylogenetic analyses in the present study revealed with strong support that it forms a distinct clade with the voucher (RAS VV11.1) of an unnamed species and that it is closer to subgenera *Paa* and *Nanorana*, although the phylogenetic relationships between these three clades were not resolved in this study. This finding is consistent with the work of Hofmann et al. [84], which revealed a distinct clade that contains three vouchers (RAS VV5.1, RAS VV8.1, and RAS VV11.1) and is the sister to the clade composed of subgenera *Nanorana* and *Paa* with strong support [84]. Therefore, we consider that this clade deserves the rank of independent subgenus in the genus *Nanorana* and suggest the subgenus name *Minipaa* **subgen. nov.** to accommodate *N. minica*, which has a small body size and was once placed in *Paa*. The 16S distance between *N. minica* and the three vouchers (RAS VV5.1, RAS VV8.1, and RAS VV11.1) is very small (0.7–0.9%), implying that they are probably conspecific, pending additional morphological data. For *N. annandalii*, *N. feae*, *N. gammii*, and *N. mokokchungensis*, their phylogenetic placements have never been investigated, and currently there are no sequences from them in GenBank, so more studies are needed to address the sub-generic allocation of these four species.

The genus *Allopaa* was erected by Ohler and Dubois [20], who recovered the type species of *Allopaa* (*A. hazarensis*) as the sister-group of other Paini based on morphological data, and currently, it only contains *A. hazarensis* [12]. Most previous phylogenetic analyses involving Paini [13,21,23,26,87] did not address its phylogenetic placement, until recently, Hofmann et al. [24,25,84] and Akram et al. [27] found that *A. hazarensis* was nested in the genus *Nanorana*. However, both Hofmann et al. [24,25,84] and Akram et al. [27] did not render the taxonomic remedy. In this study, we also found with strong support that *A. hazarensis* was nested in *Nanorana* and that it was closer to *Nanorana* (*Chaparana*), rendering currently recognized *Nanorana* paraphyletic. According to Ohler and Dubois [20], *Allopaa* can be distinguished from all other genera of Paini in several features, such as a first finger that is longer than the second, blunt tips of fingers and toes, no tarsal fold, complete webbing, a dermal fringe along the fifth toe that does not reach the basis of the metatarsus, and males with an internal vocal sac and black nuptial spines scattered on the dorsal part of the metacarpal tubercle and along finger I. However, these characters are not unique to *Allopaa* and vary among *Nanorana* species. Firstly, the first finger is also longer than the second finger in most members of the subgenus *Chaparana* (*N. quadranus*, *N. taihangnica*, *N. unculuanus*, *N. kangxianensis*, *N. sichuanensis*, *N. yunnanensis*), in some species of subgenus *Paa*, such as *N. liebigii* and *N. xuelinensis*, and in a member of subgenus *Nanorana* (*N. parkeri*) [35,88]. Secondly, the tips of the fingers and toes are round and swollen in most species of *Nanorana*, with a few dilated to small disks [35]. Thirdly, the absence of the tarsal fold is also not unique to *Allopaa*, in that many members of the genus *Nanorana* also lack it (e.g., *N. quadranus*, *N. unculuanus*, *N. kangxianensis*, *N. conaensis*, and *N. liebigii*). Fourthly, complete webbing in *Nanorna* is very common (e.g., *N. quadranus*, *N. taihangnica*, *N. kangxianensis*, *N. sichuanensis*, *N. phrynoides*, *N. liebigii*, and *N. ventripunctata*). Fifthly, a dermal fringe along the outer edge of toe V that also does not reach the basis of the metatarsus is present in many *Nanorana* species; for instance, the fringe only reaches the base of the toe in *N. quadranus*, *N. unculuanus*, *N. yunnanensis*, *N. liebigii*, and *N. polunini*. Sixthly, an internal vocal sac also presents in some *Nanorana* species, such as *N. sichuanensis*, *N. yunnanensis*, *N. phrynoides*, *N. blanfordii*, and *N. chayuensis*. Finally, the presence of nuptial spines on the fingers also varies among *Nanorana* species; for instance, *N. kangxianensis* has nuptial spines on the first finger, *N. quadranus*, *N. taihangnica*, and *N. unculuanus* have no nuptial spines on the fingers, and *N. sichuanensis* and *N. phrynoides* have nuptial spines on fingers I and II or fingers I–III. Therefore, based on the present phylogenetic analyses and morphological comparisons, we consider that the genus *Allopaa* is invalid and suggest moving it into *Nanorana* (*Chaparana*).

## 5. Conclusions

We described a new species of *Nanorana*, *Nanorana laojunshanensis* **sp. nov.** from Mt. Laojun in northwestern Yunnan, China, based on molecular and morphological evidence. The new species belongs to the subgenus *Nanorana* and is the sister to the clade of *N. pleskei* and *N. ventripunctata*. Additionally, we revealed that *N. arunachalensis* probably does not belong to the subfamily Dicroglossinae (therefore *Nanorana*) but maybe represents a distinct genus closely related to *Ingerana* or belongs to *Ingerana*, pending more data. We suggested placing *Allopaa* into the synonymy of *Nanorana* and moving it into the subgenus *Chaparana*. We considered that *N. minica* deserves the rank of an independent subgenus in genus *Nanorana*, and allocations of subgenus were suggested for other species.

**A key to members of *Nanorana* (*Nanorana*)**

1 Tympanum absent………………………………………………………………………….2

– Tympanum present…………………………………………………………………………3

2 Nuptial pad present on base of finger I……………………………………*N. bangdaensis*

– Nuptial pad present on both fingers I and II……………………………………*N. parkeri*

3 Finger I shorter than finger II………………………………………………………*N. pleskei*

– Finger I equal to finger II……………………………………………………………………4

4 Subarticular tubercles distinct; ventral surface grayish white scattered with dark blotches………………………………………………………………….…*N. ventripunctata*

– Subarticular tubercles indistinct; lacking dark blotches on ventral surface and ventral surface of limbs yolk yellow………………………………….*N. laojunshanensis* **sp. nov.**

***Minipaa* subgen. nov.**

http://zoobank.org/urn:lsid:zoobank.org:act:F6036C62-88DD-4435-BDBC-CD9A2592E588 (accessed on 19 October 2023). 

Type species: *Nanorana minica* (Dubois, 1975)

Type locality: “Dial Bajar, au sud de Chainpur, sur la riviére Seti, Ouest-Népal”.

Diagnosis: This subgenus can be distinguished from all three other subgenera of the genus *Nanorana* by the following combination of characters according to Dubois [14] and Ohler and Dubois [20]: (1) body size small (SVL of male adults 28.5–33 mm and SVL of female adults 30.5–41 mm); (2) tips of toes obviously enlarged, twice or more than twice of the diameter of phalanges; (3) webbing very incurved between extremities of adjacent toes; (4) vocal sacs present; (5) nuptial spines large, distinct, countable, and translucent or creamy, present on fingers I and II and chest; and (6) eggs entirely whitish or creamy, without colored animal pole.

Included species: *Nanorana minica* (Dubois, 1975).

Distribution: Nepal, India, Bhutan.

Etymology: From the Latin *mini*, “small”, and from the generic name *Paa*, Dubois, 1975 (from the Tamang name paa, “frog”), in which this species was originally placed. This name refers to the small body size of this taxon.

Note: We describe it as a new subgenus because *N. minica* was recovered as the sister taxon to the clade composed of subgenus *Paa* and subgenus *Nanorana*, meaning that it deserves a rank of subgenus based on the present taxonomic framework. Additionally, according to Ohler and Dubois [20], this monotypic subgenus displays two unique characters: translucent or creamy nuptial spines and entirely whitish or creamy eggs, without colored animal pole.

## Figures and Tables

**Table 1:** Species used in phylogenetic analyses of this study.

Species	Voucher no.	Locality	16S	COI	CYTB	RAG-1
*Odorrana hosii*	USNM:Herp:586991	Yeybu Village, Tanintharyi, Myanmar	MG935960	MG935666	-	-
*Amolops mengdingensis*	KIZ20160265	Mengding, Yunnan, China	MK501808	MK501811	-	-
*Occidozyga lima*	USNM:Herp:520376	Chatthin, Sagaing, Myanmar	MG935924	MG935630	-	-
*Occidozyga lingnanica*	SYS a005585	Shenzhen, Guangdong, China	ON615075	ON615615	-	-
*Occidozyga myanhessei*	USNM:Herp:587105	Dawei, Bago, Myanmar	MG935916	MG935622	-	-
*Ingerana tenasserimensis*	CAS 205064	Myanmar	AY322302	-	-	
*Ingerana borealis*	MZMU1644	Tibet, China	MT799709	-	-	KU243100
*Fejervarya cancrivora*	HW6	Guangxi, China	EU652694	EU652694	EU652694	HM163581
*Hoplobatrachus chinensis*	THW1	Jinhua, Zhejiang, China	JX181763	JX181763	JX181763	-
*Sphaerotheca breviceps*	USNM:Herp:537466	Sagaing, Myanmar	MG935993	MG935699	-	-
*Limnonectes bannaensis*	ZNAC 21020	Yunnan, China	AY899242	AY899242	AY899242	
*Quasipaa boulengeri*	XM3632	Sichuan, China	KX645665	KX645665	KX645665	-
*Chrysopaa sternosignata*	USNM:Herp:589844	Parvan, Afghanistan	MG700155	MG699938	-	-
*Allopaa hazarensis*	9386	Pakistan	MW598397	MW603006	-	MW598465
*Nanorana arunachalensis*	ZSIS-M40	Cona, Tibet, China	MN636773	-	-	-
*Nanorana (Chaparana) aenea*	2001.0277	Yunnan, China	KR827954	KR087830	-	HM163609
*Nanorana (Chaparana) quadranus*	CIB 20060644	Sichuan, China	GQ225907	OL762449	-	HM163591
*Nanorana (Chaparana) taihangnica*	LGW-LC-001	Henan, China	KF199146	KF199146	KF199146	-
*Nanorana (Chaparana) yunnanensis*	STJW-LP-001	Yunnan, China	KF199150	KF199150	KF199150	HM163592
*Nanorana (Paa) chayuensis*	MNU20190419	Chayu, China	MN411630	MN411630	MN411630	HM163587
*Nanorana (Paa) liebigii*	SH080524-NME	Nepal	MN012113	MN012241	-	MN032536
*Nanorana (Paa) medogensis*	SYNU-XZ35	Motuo, China	MH315960	-	-	HM163590
*Nanorana rarica*	A1961-13-NME	Nepal	MN012202	MN012322		
*Nanorana (Paa) rostandi*	SYNU-1507058	Tibet, China	MH315964	-	-	MW111374
*Nanorana ercepeae*	A2017-13-NME	Nepal	MN012076	MN012212		MN032500
*Nanorana zhaoermii*	SYNU-1706063	Tibet, China	MH315958	-	-	-
*Nanorana xuelinensis*	KIZL2019013	Lancang, Yunnan, Chna	MZ416027	-	-	-
*Nanorana vicina*	WLM:NV289171	Murree, Pakistan	MW898174	-	-	-
*Nanorana unculuanus*	KizYP010	Yunnan, China	DQ118491	-	-	HM163595
*Nanorana sichuanensis*	CIBYY20080693	Sichuan, China	KU140064	-	-	-
*Nanorana polunini*	2003.3085	Pangum, Nepal	KR827957			MW111375
*Nanorana phrynoides*	CIBYN090223	Yunnan, China	KU140002	-	-	-
*Nanorana maculosa*	YNU-HU2002308	Yunnan, China	EU979835	-	-	HM163588
*Nanorana kangxianensis*	CIB1247625	Gansu, China	MZ895123	MZ895123	MZ895123	-
*Nanorana conaensis*	KizYP152	Tibet, China	DQ118513	-	-	HM163589
*Nanorana blanfordii*	SYNU-1507011	Tibet, China	MH315963	-	-	MW111370
*Nanorana arnoldi*	YNU-HU200109006	Yunnan, China	EU979837	-	-	-
*Nanorana minica*	WT008	Himachal Pradesh, India	OQ079488	-	-	-
*Nanorana* sp.	VV11.1-RAS	-	OP173783	OP174426	-	OP204887
*Nanorana (Nanorana) bangdaensis*	N7_06_NME	Nimu, Lasa, Tibet, China	MN012126	MN012249	-	MN032549
*Nanorana (Nanorana) bangdaensis*	CAS802L	Dangxiong, Lasa, Tibet, China	MN012138	MN012261	-	MN032555
*Nanorana (Nanorana) bangdaensis*	KIZ014955	Bangda, Baxoi, Tibet, China	-	KJ811447	-	-
*Nanorana (Nanorana) bangdaensis*	YPX32289	Daritang, Uzi, Tibet	-	KJ811502	-	-
*Nanorana (Nanorana) bangdaensis*	YPX18274	Lulang, Nyingchi, Tibet	-	KJ811344	-	-
*Nanorana (Nanorana) bangdaensis*	YPX15383	Lhunze, Tibet	-	KJ811253	-	-
*Nanorana (Nanorana) bangdaensis*	N3_06_NME	Seni, Naqu, Tibet, China	MN012150	MN012272	-	MN032561
*Nanorana (Nanorana) bangdaensis*	KP317482	Dangxiong, Lasa, Tibet, China	KP317482	KP317482	KP317482	-
*Nanorana (Nanorana) bangdaensis*	KIZ20181001	Bangda, Baxoi, Tibet	OR678588	OR671665	-	OR678566
*Nanorana (Nanorana) bangdaensis*	KIZ20181002	Bangda, Baxoi, Tibet	OR678589	OR671666	-	OR678567
*Nanorana (Nanorana) bangdaensis*	KIZ20181003	Bangda, Baxoi, Tibet	OR678590	OR671667	-	OR678568
*Nanorana (Nanorana) bangdaensis*	KIZ20181004	Bangda, Baxoi, Tibet	OR678591	OR671668	-	OR678569
*Nanorana (Nanorana) bangdaensis*	KIZ20181005	Bangda, Baxoi, Tibet	OR678592	OR671669	-	OR678570
*Nanorana (Nanorana) parkeri*	KIZ20181101	Jilong, Rikaze, Tibet, China	OR678593	OR671670	-	OR678571
*Nanorana (Nanorana) parkeri*	KIZ20181102	Jilong, Rikaze, Tibet, China	OR678594	OR671671	-	OR678572
*Nanorana (Nanorana) parkeri*	KIZ20181103	Jilong, Rikaze, Tibet, China	OR678595	OR671672	-	OR678573
*Nanorana (Nanorana) parkeri*	KIZ20181104	Jilong, Rikaze, Tibet, China	OR678596	OR671673	-	OR678574
*Nanorana (Nanorana) parkeri*	KIZ20181105	Jilong, Rikaze, Tibet, China	OR678597	OR671674	-	OR678575
*Nanorana (Nanorana) parkeri*	KIZ08139	Gangga, Tingri, Tibet, China	-	KJ811162	-	-
*Nanorana (Nanorana) parkeri*	YPX14998	Nierixiong, Rikaze, Tibet	-	KJ811364	-	-
*Nanorana (Nanorana) parkeri*	YPX15060	Saga, Tibet	-	KJ811396	-	-
*Nanorana (Nanorana) parkeri*	YPX14646	Nierixiong, Rikaze, Tibet, China	-	KY172326	KY172509	-
*Nanorana (Nanorana) pleskei*	YPX32958	Maqu, Ganan, Gansu, China	-	KY172146	KY172329	KY172539
*Nanorana (Nanorana) pleskei*	YPX25853	Banma, Guoluo, Qinghai, China	-	KY172155	KY172338	KY172541
*Nanorana (Nanorana) pleskei*	KIZ014906	Jiangda, Changdu, Tibet	-	KY172183	KY172366	KY172552
*Nanorana (Nanorana) pleskei*	YPX32403	Kangding, Sichuan, China	-	KY172218	KY172401	KY172605
*Nanorana (Nanorana) pleskei*	KQ13_14_NME	Ganzi, Sichuan, China	MN012160	MN012282	-	MN032566
*Nanorana (Nanorana) pleskei*	KQ47_14_NME	Kangding, Ganzi, Sichuan, China	MN012156	MN012278	-	MN032562
*Nanorana (Nanorana) pleskei*	CIB20080515-1	Shiqu, Sichuan, China	HQ324232	HQ324232	HQ324232	-
*Nanorana (Nanorana) ventripunctata*	GXNU YU130021	Xiaozhongdian, Zhongdian, Yunnan, China	OR678598	OR671675	OR678554	OR678576
*Nanorana (Nanorana) ventripunctata*	GXNU YU130022	Xiaozhongdian, Zhongdian, Yunnan, China	OR678599	OR671676	OR678555	OR678577
*Nanorana (Nanorana) ventripunctata*	GXNU YU090163	Bitahai, Zhongdian, Yunnan, China	OR678600	OR671677	OR678556	OR678578
*Nanorana (Nanorana) ventripunctata*	GXNU YU000498	Bitahai, Zhongdian, Yunnan, China	OR678601	OR671678	OR678557	OR678579
*Nanorana (Nanorana) ventripunctata*	GXNU YU000502	Bitahai, Zhongdian, Yunnan, China	OR678602	OR671679	OR678558	OR678580
*Nanorana (Nanorana) ventripunctata*	GXNU YU000503	Bitahai, Zhongdian, Yunnan, China	OR678603	OR671680	OR678559	OR678581
*Nanorana (Nanorana) laojunshanensis* **sp. nov.**	GXNU YU090312	Mt. Laojun, Lijiang, Yunnan, China	OR678604	OR671681	OR678560	OR678582
*Nanorana (Nanorana) laojunshanensis* **sp. nov.**	GXNU YU090313	Mt. Laojun, Lijiang, Yunnan, China	OR678605	OR671682	OR678561	OR678583
*Nanorana (Nanorana) laojunshanensis* **sp. nov.**	GXNU YU090314	Mt. Laojun, Lijiang, Yunnan, China	OR678606	OR671683	OR678562	OR678584
*Nanorana (Nanorana) laojunshanensis* **sp. nov.**	GXNU YU090315	Mt. Laojun, Lijiang, Yunnan, China	OR678607	OR671684	OR678563	OR678585
*Nanorana (Nanorana) laojunshanensis* **sp. nov.**	GXNU YU090316	Mt. Laojun, Lijiang, Yunnan, China	OR678608	OR671685	OR678564	OR678586
*Nanorana (Nanorana) laojunshanensis* **sp. nov.**	GXNU YU090317	Mt. Laojun, Lijiang, Yunnan, China	OR678609	OR671686	OR678565	OR678587

**Table 2:** Primers used for PCR amplification and sequencing in this study.

Locus	Primer Name	Primer Sequence	Source
16S	16Sar	5′-CGCCTGTTTATCAAAAACAT-3′	[38]
	16Sbr	5′-CCGGTCTGAACTCAGATCACGT-3′	[38]
cytb	CytbF	5′-ACACCCGCCAATTTGTCTTC-3′	This study
	CytbR	5′-TGAGAGGGGAAGGAGAAGGA-3′	This study
COI	Chmf4	5′-TYTCWACWAAYCAYAAAGAYATCGG-3′	[39]
	Chmr4	5′-ACYTCRGGRTGRCCRAARAATCA-3′	[39]
RAG1	L-RAG1Ran	5′-CTGGTCGTCAGATCTTTCAGC-3′	[40]
	H-RAG1Ran	5′-GCAAAACGTTGAGAGTGATAAC-3′	[40]

**Table 3:** Best partition scheme and models estimated by the partition finder analysis for the combined data.

Subset	Partitions	Model
1	16S rRNA	GTR + I + G
2	COI_pos1	SYM + I
3	COI_pos2	HKY + I
4	COI_pos3	GTR + G
5	cytb_pos1	SYM + G
6	cytb_pos2	HKY + I
7	cytb_pos3	GTR + G
8	Rag1_pos1	GTR + G
9	Rag1_pos2	GTR + I
10	Rag1_pos3	GTR + G

**Table 4:** Genetic distances (%) between members of the subgenus *Nanorana* estimated from 16S (lower triangle) and COI sequences (upper triangle).

	Species	1	2	3	4	5
1	*N. laojunshanensis* **sp. nov.**		7.4	8.7	10.6	10.0
2	*N. ventripunctata*	1.6		8.8	11.2	10.9
3	*N. pleskei*	1.6	1.9		12.9	12.4
4	*N. bangdaensis*	2.0	2.1	2.8		3.4
5	*N. parkeri*	1.6	1.8	2.7	1.0	

**Table 5:** Measurements (in mm) of *Nanorana (Nanorana) laojunshanensis* **sp. nov.** from the type locality (M: male; F: female).

Character	GXNU YU090314	GXNU YU090313	GXNU YU090315	GXNU YU090316	GXNU YU090317	GXNU YU090312
Sex	M	M	M	M	M	F
SVL (snout–vent length)	36.1	38.5	35.9	34.9	33.3	42.9
HL (head length)	10.7	11.0	10.6	10.2	10.1	11.4
HW (head width)	11.3	12.9	11.9	11.5	10.7	13.6
SL (snout length)	4.8	4.9	5.0	4.5	4.5	5.0
IND (internarial distance)	2.3	2.4	2.3	2.1	2.1	2.7
IOD (interorbital distance)	1.9	2.2	2.1	1.9	1.8	2.3
UEW (upper eyelid width)	2.4	2.4	2.0	2.0	1.9	2.6
ED (eye diameter)	3.9	4.5	3.9	3.8	3.9	4.6
TD (tympanum diameter)	1.3	1.8	1.2	1.1	1.2	1.5
DNE (nostril–eye distance)	1.7	2.1	1.7	1.6	1.6	2.1
FHL (forearm and hand length)	15.1	15.1	15.4	16.2	13.8	17.4
TL (tibia length)	15.3	15.8	15.7	15.5	14.2	16.9
TFL (length of foot and tarsus)	26.5	27.6	26.2	27.3	24.1	30.7
FL (foot length)	18.9	20.8	18.9	20.1	17.1	22.4

**Table 6:** Factor loading of first two principal components of 13 size-adjusted morphometric characteristics of *N. laojunshanensis* **sp. nov.**/*N. ventripunctata* and of *N. laojunshanensis* **sp. nov.**/*N. pleskei*.

	*N. laojunshanensis* sp. nov. and *N. ventripunctata*		*N. laojunshanensis* sp. nov. and *N. pleskei*	
Character	PC1	PC2	PC1	PC2
Eigenvalue	4.346	3.283	6.272	3.403
% variation	33.43%	25.25%	48.24%	26.18%
HL	0.742	−0.123	−0.552	0.711
HW	0.878	−0.015	0.383	0.810
SN	0.384	−0.574	−0.411	0.783
IND	0.126	0.563	−0.840	0.289
IOD	0.677	−0.297	0.753	−0.109
UEW	−0.249	0.729	−0.800	0.320
ED	0.251	−0.755	−0.89	0.698
TD	−0.09	0.162	−0.638	0.346
NED	0.390	−0.733	−0.680	0.224
FHL	0.600	0.650	0.871	0.321
TL	0.629	0.596	0.770	0.605
TFL	0.838	0.191	0.854	0.418
FL	0.818	0.230	0.884	0.359

**Table 7:** Suggested assignment of subgenus for *Nanorana* species. “Yes” means that the species belongs to the corresponding subgenus.

Species	Suggested Subgenus				
	Subg. *Nanorana*	Subg. *Paa*	Subg. *Chaparana*	Subg. *Minipaa*	Unknown
*N. aenea*			yes		
*N. annandalii*					yes
*N. arnoldi*		yes			
*N. bangdaensis*	yes				
*N. blanfordii*		yes			
*N. chayuensis*		yes			
*N. conaensis*		yes			
*N. ercepeae*		yes			
*N. feae*					yes
*N. gammii*					yes
*N. hazarensis*			yes		
*N. kangxianensis*			yes		
*N. liebigii*		yes			
*N. maculosa*		yes			
*N. medogensis*		yes			
*N. minica*				yes	
*N. mokokchungensis*					yes
*N. parkeri*	yes				
*N. phrynoides*			yes		
*N. pleskei*	yes				
*N. polunini*		yes			
*N. quadranus*			yes		
*N. rarica*		yes			
*N. rostandi*		yes			
*N. sichuanensis*			yes		
*N. taihangnica*			yes		
*N. unculuanus*			yes		
*N. ventripunctata*	yes				
*N. vicina*		yes			
*N. xuelinensis*			yes		
*N. yunnanensis*			yes		
*N. zhaoermii*		yes			
*N. laojunshanensis* **sp. nov.**	yes				

## Data Availability

Sequence data used in this study are deposited in GenBank (https://www.ncbi.nlm.nih.gov/genbank/) (accessed on 17 October 2023).

## Supplementary Materials

The following supporting information can be downloaded at: https://www.mdpi.com/article/10.3390/ani13213427/s1, Table S1: Measurements (in mm) of *N. pleskei* collected from Xinduqiao, Sichuan, China; Table S2: Measurements (in mm) of *N. ventripunctata*.

## References

[1] Xing Y.W., Ree R.H. (2017). Uplift-driven diversification in the Hengduan Mountains, a temperate biodiversity hotspot. Proc. Natl. Acad. Sci. USA.

[2] He K., Gutiérrez E.E., Heming N.M., Koepfli K.P., Wan T., He S.W., Jin W., Liu S.Y., Jiang X.L. (2019). Cryptic phylogeographic history sheds light on the generation of species diversity in sky-island mountains. J. Biogeogr..

[3] López-Pujol J., Zhang F.M., Sun H.Q., Ying T.S., Ge S. (2011). Mountains of Southern China as “Plant Museums” and “Plant Cradles”: Evolutionary and conservation insights. Mt. Res. Dev..

[4] Myers N., Mittermeier R.A., Mittermeier C.G., Da Fonseca G.A.B., Kent J. (2000). Biodiversity hotspots for conservation priorities. Nature.

[5] Mittermeier R.A., Turner W.R., Larsen F.W., Brooks T.M., Gascon C., Zachos F.E., Habel J.C. (2011). Global biodiversity conservation: The critical role of hotspots. Biodiversity Hotspots: Distribution and Protection of Conservation Priority Areas.

[6] Zhao E.M., Yang D.T. (1997). Amphibians and Reptiles of the Hengduan Mountains Region.

[7] Liu X.L., He Y.H., Wang Y.F., Beukema W., Hou S.B., Li Y.C., Che J., Yuan Z.Y. (2021). A new frog species of the genus *Odorrana* (Anura: Ranidae) from Yunnan, China. Zootaxa.

[8] Zhang D.R., Liu S., Zhang L.X., Hui H., Xiao H., Rao D.Q. (2021). A New Species of *Glyphoglossus* Günther, 1869 (Anura: Microhylidae) from Western Yunnan, China. Asian Herpetol. Res..

[9] Rao D., Zhu J.G., Rao D.Q. (2022). Atlas of Wildlife in Southwest China: Amphibian. Atlas of Wildlife in Southwest China: Amphibian.

[10] Wu Y.H., Yan F., Stuart B.L., Prendini E., Suwannapoom C., Dahn H.A., Zhang B.L., Cai H.X., Xu Y.B., Jiang K. (2020). A combined approach of mitochondrial DNA and anchored nuclear phylogenomics sheds light unrecognized diversity, phylogeny, and historical biogeography of the torrent frogs, genus *Amolops* (Anura: Ranidae). Mol. Phylogent. Evol..

[11] Günther A.C.L.G. (1896). Report on the collections of reptiles, batrachians and fishes made by Messrs Potanin and Berezowski in the Chinese provinces Kansu and Sze-chuen. Annuaire du Musée Zoologique de l’Academie Impériale des Sciences de St. Pétersbourg.

[12] Frost D.R. Amphibian Species of the World: An Online Reference. Version 6.2. 2023. American Museum of Natural History, New York, USA. https://amphibiansoftheworld.amnh.org/index.php.

[13] Che J., Zhou W.W., Hu J.S., Papenfuss T.J., Wake D.B., Zhang Y.P. (2010). Spiny frogs (Paini) illuminate the history of the Himalayan region and Southeast Asia. Proc. Natl. Acad. Sci. USA.

[14] Dubois A. (1975). Un nouveau sous-genre (*Paa*) et trois nouvelles espèces du genre *Rana*. Remarques sur la phylogénies des Ranidés (Amphibiens, Anoures). Bull. Mus. Natl. Hist. Nat. Paris. Ser. 3 Zool..

[15] Bourret R. (1939). Notes herpétologiques sur l’Indochine française. XVII. Reptiles et batraciens reçus au Laboratoire des Sciences Naturelles de l’Université au cors de l’année 1938. Descriptions de trois espèces nouvelles. Annexe au Bulletin Général de l’Instruction Publique (Hanoi).

[16] Roelants K., Jiang J.P., Bossuyt F. (2004). Endemic ranid (Amphibia: Anura) genera in southern mountain ranges of the Indian subcontinent represent ancient frog lineages: Evidence from the molecular data. Mol. Phylogenet. Evol..

[17] Jiang J.P., Dubois A., Ohler A., Tillier A., Chen X.H., Xie F., Stöck M. (2005). Phylogenetic relationships of the tribe Paini (Amphibia, Anura, Ranidae) based on partial sequences of mitochondrial 12s and 16s rRNA genes. Zool. Sci..

[18] Chen L.Q., Murphy R.W., Lathrop A., Ngo A., Orlov N.L., Ho C.T., Somorjai I. (2005). Taxonomic chaos in Asian ranid frogs: An initial phylogenetic resolution. Herpetol. J. Lond..

[19] Frost D.R., Grant T., Faivovich J., Bain R.H., Haas A., Haddad C.F.B., de Sá R.O., Channing A., Wilkinson M., Donnellan S.C. (2006). The amphibian tree of life. Bull. Am. Mus. Nat. Hist..

[20] Ohler A., Dubois A. (2006). Phylogenetic relationships and generic taxonomy of the tribe Paini (Amphibia, Anura, Ranidae, Dicroglossinae) with diagnoses of two new genera. Zoosystema.

[21] Che J., Hu J.S., Zhou W.W., Murphy R.W., Papenfuss T.J., Chen M.Y., Rao D.Q., Li P.P., Zhang Y.P. (2009). Phylogeny of the Asian spiny frog tribe Paini (Family Dicroglossidae) sensu Dubois. Mol. Phylogenet. Evol..

[22] Qi S., Zhou Z.Y., Lu Y.Y., Li J.L., Qin H.H., Hou M., Zhang Y., Ma J.Z., Li P.P. (2019). A new species of *Nanorana* (Anura: Dicroglossidae) from southern Tibet, China. Russ. J. Herpetol..

[23] Hofmann S., Baniya C.B., Litvinchuk S.N., Miehe G., Li J.T., Schmidt J. (2019). Phylogeny of spiny frogs *Nanorana* (Anura: Dicroglossidae) supports a Tibetan origin of a Himalayan species group. Ecol. Evol..

[24] Hofmann S., Masroor R., Jablonski D. (2021). Morphological and molecular data on tadpoles of the westernmost Himalayan spiny frog *Allopaa hazarensis* (Dubois & Khan, 1979). ZooKeys.

[25] Hofmann S., Jablonski D., Litvinchuk S.N., Masroor R., Schmidt J. (2021). Relict groups of spiny frogs indicate Late Paleogene-Early Neogene trans-Tibet dispersal of thermophile faunal elements. PeerJ.

[26] Liu S., Zhang P., Rao D. (2021). A new species of *Nanorana* Günther, 1896 (Anura, Dicroglossidae) from Yunnan, China. ZooKeys.

[27] Akram A., Rais M., Lopez-Hervas K., Tarvin R.D., Saeed M., Bolnick D.I., Cannatella D.C. (2021). An insight into molecular taxonomy of bufonids, microhylids, and dicroglossid frogs: First genetic records from Pakistan. Ecol. Evol..

[28] Dufresnes C., Litvinchuk S.N. (2022). Diversity, distribution and molecular species delimitation in frogs and toads from the Eastern Palaearctic. Zool. J. Linn. Soc..

[29] Shrestha B., Suwal S.P., Pandey B., Das J., Manandhar P., Karmacharya D., Ohler A., Dubois A., O’Connell K.A. (2022). Molecular and morphological identification of frog species collected at Rara Lake in Rara National Park, Nepal. Zootaxa.

[30] Dubois A., Ohler A., Pyron R.A. (2021). New concepts and methods for phylogenetic taxonomy and nomenclature in zoology, exemplified by a new ranked cladonomy of recent amphibians (Lissamphibia). Megataxa.

[31] Dubois A. (1992). Notes sur la classification des Ranidae (Amphibiens anoures). Bull. Mens. Soc. Linn. Lyon..

[32] Stejneger L. (1927). A new genus and species of frog from Tibet. J. Wash. Acad. Sci..

[33] Fei L., Huang Y.Z. (1985). A new species of the genus *Nanorana* (Amphibia: Ranidae) from northwestern Yunnan, China. Acta Biol. Plateau Sin..

[34] Fei L., Ye C.Y., Jiang J.P. (2010). Colored Atlas of Chinese Amphibians.

[35] AmphibiaChina (2023). The Database of Chinese Amphibians. Kunming Institute of Zoology (CAS), Kunming, Yunnan, China. http://www.amphibiachina.org/.

[36] Fei L., Ye C.Y., Jiang J.P., Xie F. (2005). An Illustrated Key to Chinese Amphibians.

[37] Fei L., Hu S.Q., Ye C.Y., Huang Y.Z. (2009). Fauna Sinica, Amphibia, Vol. 3 Anura Ranidae.

[38] Palumbi S.R., Martin A., Romano S., Owen MacMillan W., Stice L., Grabowski G. (1991). The Simple Fool’s Guide to PCR.

[39] Che J., Chen H.M., Yang J.X., Jin J.Q., Jiang K., Yuan Z.Y., Murphy R.W., Zhang Y.P. (2012). Universal COI primers for DNA barcoding amphibians. Mol. Ecol. Resour..

[40] Stuart B.L. (2008). The phylogenetic problem of *Huia* (Amphibia: Ranidae). Mol. Phylogenet. Evol..

[41] Murray J.A. (1885). A new frog (*Rana sternosignata*) from Sind. Ann. Mag. Nat. Hist. Ser. 5.

[42] Günther A.C.L.G. (1889). Third contribution to our knowledge of reptiles and fishes from the upper Yangtze-Kiang. Ann. Mag. Nat. Hist. Ser. 6.

[43] Dubois A., Khan M.S. (1979). A new species of frog (genus *Rana*, subgenus *Paa*) from northern Pakistan (Amphibia, Anura). J. Herpetol..

[44] Gravenhorst J.L.C. (1829). Deliciae Musei Zoologici Vratislaviensis. Fasciculus primus. Chelonios et Batrachia.

[45] Lyu Z.T., Wang J., Zeng Z.C., Luo L., Zhang Y.W., Guo C.P., Ren J.L., Qi S., Mo Y.M., Wang Y.Y. (2022). Taxonomic clarifications on the floating frogs (Anura: Dicroglossidae: *Occidozyga* sensu lato) in southeastern China. Vertebr. Zool..

[46] Köhler G., Vargas J., Than N.L., Schell T., Janke A., Pauls S.U., Thammachoti P. (2021). A taxonomic revision of the genus *Phrynoglossus* in Indochina with the description of a new species and comments on the classification within Occidozyginae (Amphibia, Anura, Dicroglossidae). Vertebr. Zool..

[47] Annandale N. (1912). Zoological results of the Abor Expedition, 1911–1912. I. Amphibia. Rec. Indian Mus..

[48] Sclater W.L. (1892). On some specimens of frogs in the Indian Museum, Calcutta with description of several new species. Proc. Zool. Soc. Lond..

[49] Osbeck P. (1765). Reise nach Ostindien und China. Nebst O. Toreens Reise nach Suratte und C. G. Ekebergs nachricht von der Landwirthschaft der Chinesen. Aus dem Schwedischen Übersetzt von J. G. Georgi.

[50] Schneider J.G. (1799). Historia Amphibiorum Naturalis et Literarariae. Fasciculus Primus. Continens Ranas, Calamitas, Bufones, Salamandras et Hydros in Genera et Species Descriptos Notisque suis Distinctos.

[51] Yu G.H., Wu Z.J., Yang J.X. (2019). A new species of the *Amolops monticola* group (Anura: Ranidae) from southwestern Yunnan, China. Zootaxa.

[52] Boulenger G.A. (1891). On new or little-known Indian and Malayan reptiles and batrachians. Ann. Mag. Nat. Hist. Ser. 6.

[53] Kumar S., Stecher G., Tamura K. (2016). MEGA7: Molecular Evolutionary Genetics Analysis version 7.0 for bigger datasets. Mol. Biol. Evol..

[54] Lanfear R., Frandsen P.B., Wright A.M., Senfeld T., Calcott B. (2016). PartitionFinder 2: New methods for selecting partitioned models of evolution formolecular and morphological phylogenetic analyses. Mol. Biol. Evol..

[55] Lanfear R., Calcott B., Ho S.Y.W., Guindon S. (2012). PartitionFinder: Combined selection of partitioning schemes and substitution models for phylogenetic analyses. Mol. Biol. Evol..

[56] Ronquist F., Teslenko M., van der Mark P., Ayres D.L., Darling A., Höhna S., Larget B., Liu L., Suchard M.A., Huelsenbeck J.P. (2012). MrBayes 3.2: Efficient Bayesian phylogenetic inference and model choice across a large model space. Syst. Biol..

[57] Edler D., Klein J., Antonelli A., Silvestro D. (2021). raxmlGUI 2.0: A graphical interface and toolkit for phylogenetic analyses using RAxML. Methods Ecol. Evol..

[58] Saikia B., Sinha B., Kharkongor I. (2017). *Odorrana arunachalensis*: A new species of Cascade Frog (Anura: Ranidae) from Talle Valley Wildlife Sanctuary, Arunachal Pradesh, India. J. Bioresour..

[59] Smith M.A. (1922). Notes on reptiles and batrachians from Siam and Indo-China (No. 1). J. Nat. Hist. Soc. Siam.

[60] Boulenger G.A. (1920). A monograph of the South Asian, Papuan, Melanesian and Australian frogs of the genus *Rana*. Rec. Indian Mus..

[61] Anderson J. (1871). A list of the reptilian accession to the Indian Museum, Calcutta from 1865 to 1870, with a description of some new species. J. Asiatic Soc. Bengal.

[62] Günther A.C.L.G. (1860). Contribution to the knowledge of the reptiles of the Himalaya mountains. Proc. Zool. Soc. Lond..

[63] Smith M.A. (1951). On a collection of amphibians and reptiles from Nepal. Ann. Mag. Nat. Hist. Ser. 12.

[64] Dubois A., Matsui M., Ohler A. (2001). A replacement name for *Rana (Paa) rara* Dubois & Matsui, 1983 (Amphibia, Anura, Ranidae, Raninae). Alytes.

[65] Dubois A. (1974). Diagnoses de trois espèces nouvelles d’amphibiens du Népal. Bull. Soc. Zool. Fr..

[66] Liu C.C., Hu S.Q., Yang F.H. (1960). Amphibia of Yunnan collected in 1958. Acta Zool. Sin..

[67] Stoliczka F. (1872). Notes on some new species of Reptilia and Amphibia, collected by Dr. W. Waagen in North-western Punjab. Proc. Asiat. Soc. Bengal.

[68] Anderson J. (1879). Anatomical and Zoological Researches: Comprising an Account of the Zoological Results of the Two Expeditions to Western Yunnan in 1868 and 1875; and a Monograph of the Two Cetacean Genera Platanista and Orcella.

[69] Boulenger G.A. (1882). Catalogue of the Batrachia Salientia s. Ecaudata in the Collection of the British Museum.

[70] Ye C.C. (1977). A survey of amphibians in Xizang (Tibet). Acta Zool. Sin..

[71] Huang Y.Z., Fei L. (1981). Two new species of amphibians from Xizang. Acta Zootaxonomica Sin..

[72] Boulenger G.A. (1887). An account of the batrachians obtained in Burma by M.L. Fea of the Genoa Civic Museum. Ann. Mus. Civico Storia Nat. Genova Ser. 2.

[73] Fei L. (1999). Atlas of Amphibians of China.

[74] Das I., Chanda S.K. (2000). A new species of *Scutiger* (Anura: Megophryidae) from Nagaland, north-eastern India. Herpetol. J. Lond..

[75] Boulenger G.A. (1917). Descriptions of new frogs of the genus *Rana*. Ann. Mag. Nat. Hist. Ser. 8.

[76] Dubois A. (1987). Miscellanea taxinomica batrachologica (I). Alytes.

[77] Yang X., Wang B., Hu J.H., Jiang J.P. (2011). A new species of the genus *Feirana* (Amphibia: Anura: Dicroglossidae) from the Western Qinling Mountains of China. Asian Herpetol. Res..

[78] Liu C.C., Hu S.Q., Yang F.H. (1960). Amphibians from Wushan, Szechwan. Acta Zool. Sin..

[79] Chen X.H., Jiang J.P. (2002). A new species of the genus *Paa* from China. Herpetol. Sin..

[80] Zhou W.W., Zhang B.L., Chen H.M., Jin J.Q., Yang J.X., Wang Y.Y., Jiang K., Murphy R.W., Zhang Y.P., Che J. (2014). DNA barcodes and species distribution models evaluate threats of global climate changes to genetic diversity: A case study from *Nanorana parkeri* (Anura: Dicroglossidae). PLoS ONE.

[81] Wang G.D., Zhang B.L., Zhou W.W., Li Y.X., Jin J.Q., Shao Y., Yang H.C., Liu Y.H., Yan F., Chen H.M. (2018). Selection and environmental adaptation along a path to speciation in the Tibetan frog *Nanorana parkeri*. Proc. Natl. Acad. Sci. USA.

[82] Vieites D.R., Wollenberg K.C., Andreone F., Köhler J., Glaw F., Vences M. (2009). Vast underestimation of Madagascar’s biodiversity evidenced by an integrative amphibian inventory. Proc. Natl. Acad. Sci. USA.

[83] Tang S.J., Sun T., Liu S., Luo S.D., Yu G.H., Du L.N. (2023). A new species of cascade frog (Anura: Ranidae: Amolops) from central Yunnan, China. Zool. Lett..

[84] Hofmann S., Schmidt J., Masroor R., Borkin L.J., Litvintchuk S., Rödder D., Vershinin V., Jablonski D. (2023). Endemic lineages of spiny frogs demonstrate the biogeographic importance and conservational needs of the Hindu Kush-Himalaya region. Zool. J. Linn. Soc..

[85] Zug G.R. (2022). Amphibians and Reptiles of Myanmar: Checklists and Keys I. Amphibians, Crocodilians, and Turtles. Smithson. Contrib. Zool..

[86] Wangyal J.T., Norbu L., Ghalley T.B., Shacha N. (2023). First record of the Arunachal cascade frog, *Nanorana arunachalensis* (Saikia et al., 2017), from the Himalayan Kingdom of Bhutan. Herpetol. Notes.

[87] Pyron R.A., Wiens J.J. (2011). A large-scale phylogeny of Amphibia including over 2800 species, and a revised classification of advanced frogs, salamanders, and caecilians. Mol. Phylogenet. Evol..

[88] Che J., Jiang K., Yang F., Zhang Y.P. (2020). Amphibians and Reptiles in Tibet: Diversity and Evolution.

