# Supplementary material for: A New Species of Nanorana (Anura: Dicroglossidae) from Northwestern Yunnan, China, with Comments on the Taxonomy of Nanorana arunachalensis and Allopaa
Source: Animals (Basel). 2023 Nov 6;13(21):3427. doi: 10.3390/ani13213427 (PMC10649098; doi:10.3390/ani13213427)
Supplement: Supplementary file 1 [file animals-13-03427-s001.zip › animals-2585586-supplementary.pdf]

animals-2585586-supplementary file 1

Support information

Specimens of other *Nanorana (Nanorana)* species examined in this study

A total of five specimens of *Nanorana bangdaensis* (KIZ20181001–20181005), eight specimens of *Nanorana parkeri* (KIZ20181101–20181108), eight specimens of *Nanorana pleskei* (KIZ 008807–008814), and eight specimens of *Nanorana ventripunctata* (GXNU YU130019–130022, YU090163, YU000498, YU000502, and YU000503) were examined. The specimens of *N. bangdaensis* were collected from Bangda, Baxoi, Tibet, China and were deposited at Kunming Institute of Zoology, Chinese Academy of Sciences (KIZ). The specimens of *N. parkeri* were collected from Jilong, Rikaze, Tibet, China and were deposited at KIZ. The specimens of *N. pleskei* were collected form Xinduqiao, Sichuan, China and were deposited at KIZ. The specimens of *N. ventripunctata* were collected from Xiaozhongdian (GXNU YU130019–130022) and Bitahai (GXNU YU090163, GXNU YU000498, GXNU YU000502, and GXNU YU000503), Yunnan, China and were deposited at Guangxi Normal University (GXNU).

Nanorana bangdaensis:

Males: KIZ20181001, KIZ20181003, KIZ20181004, KIZ20181005.

Female: KIZ20181002.

Nanorana parkeri:

Males: KIZ20181101, KIZ20181102, KIZ20181103, KIZ20181106.

Females: KIZ20181104, KIZ20181105, KIZ20181107, KIZ20181108.

Nanorana pleskei:

Males: KIZ008807, KIZ008808, KIZ008809, KIZ008810.

Females: KIZ008811, KIZ008812, KIZ008813, KIZ008814.

Nanorana ventripunctata:

Males: GXNU YU130021, GXNU YU130022, GXNU YU090163, GXNU YU000502, GXNU YU000503.

Females: GXNU YU130019, GXNU YU130020, GXNU YU000498.

Table S1. Measurements (in mm) of *N. pleskei* collected from Xinduqiao, Sichuan, China

Character	KIZ 008807	KIZ 008808	KIZ 008809	KIZ 008810	KIZ 008811	KIZ 008812	KIZ 008813	KIZ 008814
Sex	M	M	M	M	F	F	F	F
SVL	34.8	34.6	34.5	33.0	39.7	40.2	41.6	37.9
HL	12.0	11.1	11.6	10.6	12.6	11.9	12.8	12.0
HW	11.5	11.5	11.7	10.4	12.6	12.5	12.7	11.9
SL	4.8	5.1	5.3	4.6	5.7	5.3	5.5	5.1
IND	3.0	2.9	2.8	3.0	3.5	2.9	3.5	3.3
IOD	1.7	1.6	1.4	1.8	1.8	1.7	1.7	2.0
UEW	2.4	2.8	2.5	2.2	2.7	2.8	2.9	2.6
ED	4.4	3.9	4.2	3.8	4.6	4.5	4.4	3.8
TD	1.5	1.4	1.5	1.3	1.7	1.5	1.7	1.7
DNE	1.6	2.2	2.0	1.9	2.3	2.3	2.1	2.3
FHL	13.9	13.2	14.3	12.6	15.7	14.6	14.6	14.8
TL	14.5	14.2	14.8	13.3	15.3	14.6	14.9	14.7
TFL	24.0	24.6	24.9	22.1	26.9	24.7	25.6	25.8
FL	17.2	17.5	17.8	16.0	18.4	17.4	17.9	18.0

Table S2. Measurements (in mm) of *N. ventripunctata*

Character	GXNU YU130019	GXNU YU130020	GXNU YU130021	GXNU YU130022	GXNU YU000502	GXNU YU000503	GXNU YU090163	GXNU YU000498
Sex	F	F	M	M	M	M	M	F
SVL	47.1	42.0	36.7	35.1	43.7	35.7	42.9	49.6
HL	12.0	11.3	11.0	10.3	13.0	10.4	13.1	14.1
HW	14.5	13.1	11.7	11.5	14.5	12.1	14.5	15.5
SL	5.8	4.7	4.6	4.4	5.1	4.5	5.3	5.7
IND	2.9	2.9	2.4	2.5	3.0	2.4	2.9	3.1
IOD	2.2	2.1	1.6	1.8	2.6	1.9	2.4	2.7
UEW	3.3	3.2	2.5	2.5	2.9	2.5	3.0	2.9
ED	4.8	4.4	4.0	3.9	4.6	3.7	4.6	5.0
TD	1.8	1.6	1.6	1.6	1.8	1.3	2.0	2.2
DNE	1.9	1.7	1.6	1.7	2.0	1.5	2.1	2.2
FHL	20.0	17.5	15.9	15.4	20.1	17.1	19.3	21.2
TL	20.1	18.2	16.0	14.9	19.6	15.9	20.6	21.8
TFL	33.7	29.4	25.8	24.4	33.4	26.7	33.5	35.4
FL	23.7	22.3	18.7	18.0	24.1	19.9	24.2	25.1
